# Assessment of season-dependent body condition scores in relation to faecal glucocorticoid metabolites in free-ranging Asian elephants

**DOI:** 10.1093/conphys/cox039

**Published:** 2017-06-27

**Authors:** Sanjeeta Sharma Pokharel, Polani B. Seshagiri, Raman Sukumar

**Affiliations:** 1 Centre for Ecological Sciences, Indian Institute of Science, Bangalore 560012, Karnataka, India; 2 Department of Molecular Reproduction and Developmental Genetics, Indian Institute of Science, Bangalore 560012, Karnataka, India

**Keywords:** Asian elephants, body condition score, *Elephas maximus*, faecal glucocorticoid metabolites, seasonality, stress physiology

## Abstract

We studied seasonal and annual changes in visual body condition scores (BCSs), and assessed how these scores were related to levels of faecal glucocorticoid metabolites (fGCMs) in free-ranging Asian elephants (*Elephas maximus*) in the seasonally dry tropical forests of the Mysore and Nilgiri Elephant Reserves in southern India. We assessed the animals’ BCS visually on a scale of 1 to 5; where 1 represents a very thin and 5 represents a very fat elephant. To understand the influence of seasonality on BCS, we sampled the population during dry (*n* = 398) and wet seasons (*n* = 255) of 2013 and 2015 while, for annual changes in BCS, we sampled nine free-ranging adult females from different family groups that had been repeatedly sighted over seven years. To evaluate the influence of body condition on fGCM, 307 faecal samples were collected from 261 different elephants and were analysed. As a parameter of adrenocortical activity, and thus stress, fGCM was measured (μg/g) in the ethanol-extracted samples using a group-specific 11-oxoaetiocholanolone EIA (antibody raised against 11-oxoaetiocholanolone-17-CMO:BSA and biotinylated-11-oxoaetiocholanolone as a label). Effect of age and season on BCS in relation to fGCM was also studied. A seasonal shift in BCS was observed as expected, i.e. individuals with low BCS were more frequent during the dry season when compared with the wet season. Concentrations of fGCM were highest in individuals with lowest BCS (BCS 1) and then significantly declined till BCS 3. fGCM levels were almost comparable for BCS 3, 4 and 5. This pattern was more conspicuous in female than in male elephants. Season-dependent BCS, hence, reflect the stress status as measured by fGCM, especially in female Asian elephants. This could be used as an important non-invasive approach to monitor the physiological health of free-ranging elephant populations.

## Teaser

More ‘stressed’ Asian elephants, as indicated by higher faecal glucocorticoid metabolites (fGCMs), have lower visually determined body condition scores (BCSs) that also changes seasonally. Changes in BCS are more obvious in female than in male elephants. BCS and fGCM are valuable approaches in monitoring the physiological health of free-ranging elephant populations.

## Introduction

In a natural environment, large and long-ranging herbivorous mammals such as elephants may have to face various ecological challenges or stressful conditions. One such challenge that might impact their health is resource limitation, either in terms of quality or quantity. Resource limitation for mammals due to seasonal change has been documented in tropical habitats (McNaughton *et al.*, 1988; Ahrestani *et al.*, 2012). Many Asian and African elephant populations live in seasonal environments which vary in the availability of water, forage and nutritional quality (Sukumar, 2003). Such seasonal transformation can influence the foraging behaviour or the physiology of an individual with consequences for their health, body condition and overall fitness.

Prolonged exposure to environmental perturbation or food shortage triggers stress in an individual (Wingfield *et al.*, 1998). Physiological ‘stress’, marked by the secretion of stress hormones such as glucocorticoids, helps an individual to cope up with the changing environment, ensuring survival and allowing adaptation to such changes (Moberg, 2000; Möstl and Palme, 2002; Menargues *et al.*, 2008). The Cort-fitness hypothesis suggests that if such physiological stress persists for a prolonged period, it loses its adaptive value and adversely impacts the health and longevity of the species by impairing endocrine and immune functions, degrading body mass (muscle, liver, bone and adipose tissues), individual fitness and causing reproductive failure (Munck *et al.*, 1984; Kaplan, 2000; Bonier *et al.*, 2009; Muehlenbein *et al.*, 2012).

Elevated levels of circulating glucocorticoids result in inhibition of protein synthesis, increased energy mobilization and a decrease in the maintenance of structural tissues with invariable reduction in body mass and condition (Landys *et al.*, 2006; Schakman *et al.*, 2013). The individual's reproductive potential, health status in terms of presence or absence of diseases or disorders and food availability can be determined by a reflection of its fat reserves or ‘body condition’ (Albl, 1971; Gerhart *et al.*, 1996). Baseline and elevated glucocorticoid levels have been shown to seasonally vary in many wild, free-living vertebrates (Romero, 2002). Glucocorticoid responses of an organism may be influenced by numerous factors, including individual (metabolic rate or energy expenditure, diet, gut microbes and parasites), social (group-size, dominance rank), physiological (reproductive and stress status) and ecological (predation pressures, adverse climatic conditions and food availability) factors (Wingfield *et al.*, 1998; Goymann, 2012; Cockrem, 2013; Creel *et al.*, 2013; Dantzer *et al.*, 2014; Haase *et al.*, 2016) and even their individual stress coping strategies (Koolhaas *et al.*, 1999). It is thus a reasonable assumption that an individual with poor body condition or reduced body mass (due to extreme seasonal condition or scarce resources) may have a heightened level of stress hormones. At the other extreme of the scale, an ‘obese’ body type in mammals such as rats, humans and nonhuman primates has generally been associated with increased levels of total cortisol metabolite excretion (Björntorp, 1995; Livingstone *et al.*, 2000; Jackson *et al.*, 2017). Although in these studies, the level of cortisol or hyperactivation of the hypothalamic–pituitary–adrenal axis was considered to contribute to visceral adiposity or upper body obesity, the relationship between cortisol and obesity is highly complex. The pathology of obesity depends on various signals, tissue-specific cortisol metabolism, diet, appetite-regulating hormones and gender (sex hormones), with chronically elevated cortisol levels being either a cause or a consequence of obesity (Andrew *et al.*, 1998; Björntorp and Rosmond, 2000; Bose *et al.*, 2009; Hariri and Thibault, 2010). Among elephants it may be reasonable to assume that an animal in ‘poor’ body condition because of adverse environment, nutritional deficiency or disease may have elevated glucocorticoid levels, but the relationship between ‘obesity’ and glucocorticoid levels in elephants is not clear. Large male elephants considered to be in good body condition are more likely to show prolonged expression of musth, a condition that enhances reproductive success (Sukumar, 2003).

Being a subjective visual tool to assess the amount of reserved energy stored in body fat and muscle tissues of animals and also an index of their health status, body condition scores (BCSs) can be broadly assessed by scoring the extent of visibility of bones, its concavity and depressions (Riney, 1960; Burkholder, 2000; Alapati *et al.*, 2010). Several techniques for assessing the body condition of captive and wild elephants have been developed (Albl, 1971; Wemmer *et al.*, 2006; Fernando *et al.*, 2009; Morfeld *et al.*, 2014; Wijeyamohan *et al.*, 2015). Beside this, studies have also validated the BCS with various invasive techniques of measuring kidney fat, adrenal weight, fat deposition on ribs, packed cell volume and bone marrow dry weight (Albl, 1971; Malpas, 1977; Choquenot, 1991; Gallivan and Culverwell, 1995). Most of these body condition scoring techniques rely on the ordinal scales assigned to the degree of visibility of depressions around bones and are developed for captive individuals (which makes it difficult to assign the BCS in free-ranging animals that can often be only observed safely from a distance, and not always under conditions of good visibility, light and angle) and, often, are not validated (either by measuring the fat index or by checking the reliability and repeatability). Hence, in this study, we adapted the technique defined in Morfeld *et al.* (2014, 2016) who used a simpler body condition scale of 1 (very thin) to 5 (very fat); in this method the reliability and repeatability of the visual BCS method was also checked (based on high intra- and inter-assessor agreement across multiple assessors, and biologically validated using ultrasound measures of subcutaneous fat (for captive African elephants) and serum triglyceride levels (for captive Asian elephants)). It is, hence, also a quicker method to assign BCS in free-ranging elephants as compared to the more elaborate 10-point or additive point scales used by others (Wemmer *et al.*, 2006; Fernando *et al.*, 2009; Ramesh *et al.*, 2011; Wijeyamohan *et al.*, 2015).

Several studies have demonstrated the influence of seasonality, sex, age class and injuries on hormone metabolites and BCS for African elephants (Foley *et al.*, 2001; Brown *et al.*, 2010; Ganswindt *et al.*, 2010b). Apart from a study on long-term relationship of body weights and population dynamics performed by Mumby *et al.* (2015) on captive elephants in Myanmar, there is no study demonstrating annual changes in BCS in Asian elephants. There are only a few studies on Asian elephants, either in semi-captive environment (in Myanmar) or in captivity, based on BCS and its relation with faecal glucocorticoid metabolites (fGCMs; Kumar *et al.*, 2014; Mumby *et al.*, 2015).

This study broadly aims to provide a visual description of BCS, influence of seasonality on BCS and their relationship to non-invasively sampled fGCMs that may be indicators of physiological stress in free-ranging Asian elephants. In particular, we investigated (i) annual changes in BCS over seasons and several years in a sample of adult females, (ii) dry and wet seasonal differences in BCS over two years with the expectation that body condition would be lower in the dry season and (iii) the relationship between body condition and fGCM to ascertain whether the former attribute would serve to monitor stress levels in elephants.

## Materials and methods

### Study area

Spread over an area of around 12 000 km^2^, including the Western Ghats and adjoining areas of the Eastern Ghats of southern India, the Nilgiri–Mysore–Wyanad Elephant Reserves shelter the single largest population of Asian elephants globally (Sukumar, 2003). The study was conducted primarily in the Bandipur and the Nagarahole National Parks of the Mysore Elephant Reserve, Karnataka State (Supplementary Figure 1). Located between 11°35′–11°55′N and 11°50′–12°15′N to 76°12′–76°51′E and 76°0′–76°15′E, Bandipur and Nagarahole National Parks hold about 1700 elephants in an area of 906 km^2^ and about 1300 elephants in 643 km^2^, respectively (Varma and Sukumar, 2012). With an undulating topography and altitude ranging from 260 to 1455 m a.s.l (general elevation is 800–1100 m a.s.l), the vegetation type in Bandipur is predominantly tropical dry deciduous forest along with other forest types such as moist deciduous forest, dry thorn forest and teak (*Tectona grandis*) plantations. With an average elevation of 800 m a.s.l (ranging from 959 m to 701 m a.s.l), Nagarahole includes predominantly moist deciduous forest with patches of semi-evergreen forest, dry deciduous forest, dry thorn forest, teak plantations and swampy grasslands called ‘hadlus’ (Nair *et al.*, 1980; Karanth and Sunquist, 1992). The annual average rainfall in Bandipur varies from 800 to 1300 mm along a spatial gradient (east to west), while Nagarahole similarly experiences an average annual rainfall spread of 900–1500 mm (east to west). All areas of these two National Parks were extensively surveyed in the months of February to May (dry season) and August to December (wet season) during 2013 and 2015. Beside these, some samples from identified elephants were also included from the Hassan district (an average annual rainfall of 1000 mm in the Alur Taluk and c. 1700 mm in parts of the Sakleshpur Taluk where most of the work was carried out) of Karnataka, to the north of Nagarahole, and a part of the Mysore Elephant Reserve. Longer term changes in BCS were also determined for nine identified adult female elephants in the adjoining Mudumalai National Park (Tamil Nadu State; average annual rainfall varies from 720 mm in the east to 1680 mm in the west) of the Nilgiri Elephant Reserve.

### Body condition scoring

Body condition of the sampled and non-sampled individuals were assessed visually by scoring the body fat deposition patterns around the ribs, pelvic and back bone, and an area of depression around the lumbar and pelvic regions. There are several visual BCS techniques designed for captive and free-ranging Asian elephants; a numeric scale of 10-points (Fernando *et al.*, 2009; Wijeyamohan *et al.*, 2015) or 11-points (Wemmer *et al.*, 2006) have been assigned in these methods. Most of these scoring methods rely on multiple scores, are meant for captive individuals, require summing up the individual body region scores and are not cross-validated through biological measures of adiposity. Hence, we needed a method which was validated (using ultrasound measures of actual fat thickness and other biological parameters), reliable and having fewer points scale to quickly assign the BCS to free-ranging elephants (as used in Poole, 1989; Ganswindt *et al.*, 2010b; Morfeld *et al.*, 2014, 2016).

**Figure 1:**
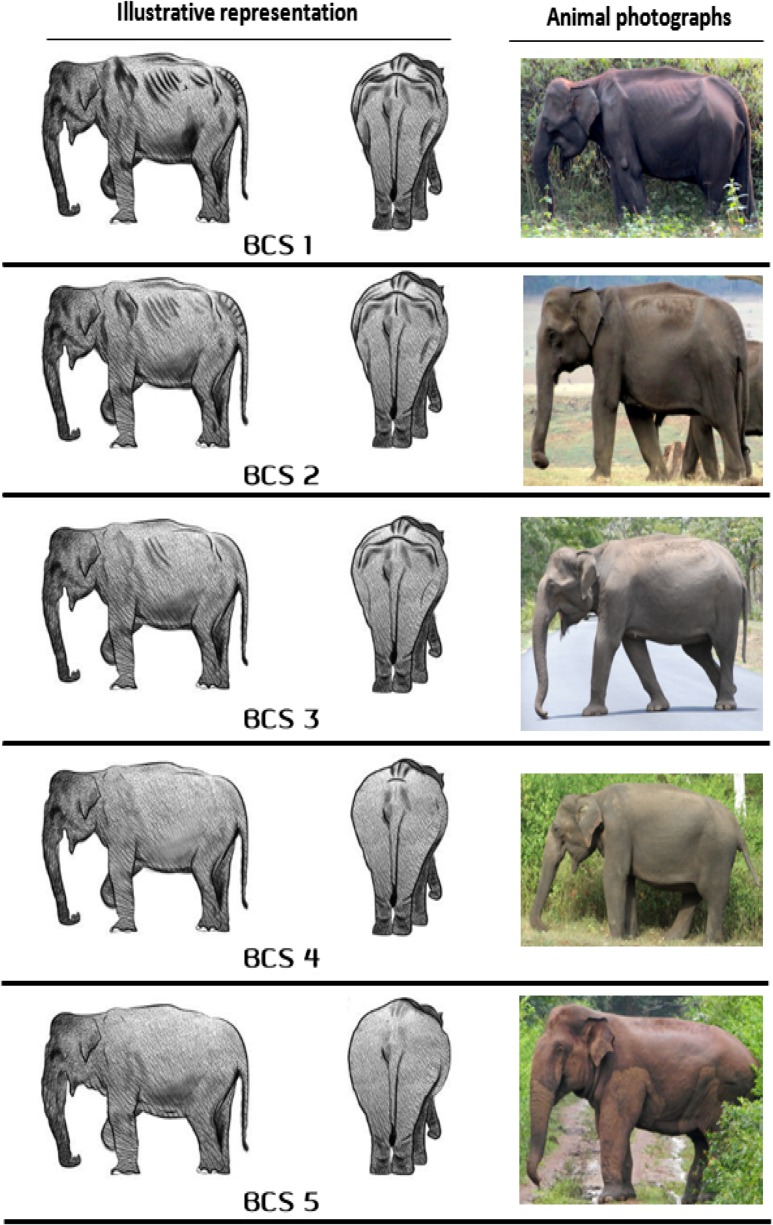
Representative schematic and photographic (animal photographs depict adult females having BCS 1 to BCS 4, and BCS 5 corresponds to a tuskless male. This latter photograph was included since we were unable to capture a high-quality photograph of a female elephant with BCS 5) illustrations of elephants showing their body conditions with assigned BCS values, ranging from 1 to 5. Criteria of assigning BCS values are described in Table 1.

For this, we adapted the protocol defined in Morfeld *et al.* (2014, 2016), that used a simpler scale of 1 (very thin) to 5 (very fat) and cross-validated it with ultrasound measures of subcutaneous fat and physiological measures of adiposity (serum triglycerides), to visually score body condition in free-ranging Asian elephants. We too scored body condition from a score of 1 being ‘very thin’ to a score of 5 being ‘very fat’ (Fig. 1 and Supplementary Table 1). We scored BCS at the time of sighting the animal and also took photographs at different angles (as described in Morfeld *et al.*, 2016) to cross-validate the scores. In total, 653 individuals were scored in two years to understand the influence of seasonality on body condition (*n* = 773); including 261 individuals for assessing hormone metabolites (*n* = 307) (Supplementary Table 2).

Several authors have also used terms such as ‘emaciated’ (for the lowest BCS), ‘normal,’ ‘optimum’ or ‘ideal’ (for mid-scale BCS) or ‘obese’ (for an extremely high BCS) to describe elephant body types (Poole, 1989; Ganswindt *et al.*, 2010b; Morfeld *et al.*, 2016). These terms may be misconstrued as value judgements about an animal's health. As we do not know the relationship between such body condition and measures of fitness such as reproduction and survivorship, we have restricted our description to the factual terms ‘very thin’, ‘thin’, ‘medium’, ‘fat’ and ‘very fat’.

#### Assessing annual changes in BCSs

To characterize annual change in BCS, the data were compiled from an ongoing project on long-term demographic monitoring of free-ranging Asian elephants in the Mudumalai National Park of Tamil Nadu State, adjoining Bandipur (in fact, many elephant groups range between Mudumalai and Bandipur). To avoid discrepancies in scoring BCS due to growth in calves, juveniles and sub-adults over the years, we selected only nine adult females from different herds which were sighted repeatedly over the seven years (2009 to 2015) of the study. Body condition was scored based on photographs (captured by Nachiketha Sharma) and was sub-grouped into two seasons based on the months of sightings.

#### Assessing seasonal changes in BCSs

The study was carried out during two broad seasons: dry season (January to May) and wet season (June to December). We further sub-classified the wet season into two categories—first wet season (from June to August when the summer or south-west monsoon is dominant) and second wet season (from September to December when the winter or north-east monsoon brings rain to this region)—to see whether the finer seasonal scale influences BCS. We sampled the populations in the Bandipur and the Nagarahole National Parks as close together in time as possible to reduce the chances that our results are an artefact of seasonal differences. A total of 653 individuals (including 261 individuals sampled for hormonal analysis) were scored for their body condition (*n* = 773), seasonally (Supplementary Table 2). These BCSs were further compared between females, males and adult individuals to see the influence of seasonality.

### Faecal sample collection

For a more comprehensive understanding of the physiological status of an individual, a longitudinal hormonal sampling over a certain period of time and biological event would be necessary. But in this study, because of practical limitations in following free-ranging elephants in a forested area with dense understorey, it was possible to carry out only cross-sectional sampling of the population, wherein we collected fresh faecal samples from identified individuals. Herds of elephants or solitary individuals (usually bulls) were followed and photographed; to avoid pseudo-replication; all the sampled individuals were identified and coded based on morphological characteristics such as cuts, folds and venation patterns in the ear-flaps; tail length and presence of tail hairs and size and orientation of the tusks in males (Sukumar, 1989; Vidya *et al.*, 2014). Additional information from characteristic morphological traits such as warts and skin folds, if any, were also recorded. Sex and age class (estimated based on shoulder height; calves (<1-year old), juveniles (1–5 years old), sub-adults (5–15 years old) and adults (above 15 years) and was re-confirmed using dung bolus circumference) of the herds of sampled individuals were noted (Sukumar, 1989; Reilly, 2002).

Elephants were observed from a safe distance until they defecated and moved away. Shortly after the defecation where possible, fresh faecal samples from the identified individuals were collected from the centre of the bolus to avoid possible contamination, labelled and stored in transportable ‘Coleman’ ice-box with ice packs at −20°C to freeze the sample in the field. The frozen samples were then transferred to a −20°C deep freezer (in order to prevent possible degradation of metabolites by bacterial enzymes) until further extraction and analysis (Möstl *et al.*, 1999; Wasser *et al.*, 2000; Möstl and Palme, 2002; Palme, 2005; Sheriff *et al.*, 2011; Palme *et al.*, 2013). To avoid bias in measuring the hormone metabolites due to contamination, mixing or any other ambient/external factors as such, we did not collect samples mixed with rain water, urine, and/or samples defecated into rivers, streams or ponds. In total, 307 fresh faecal samples were collected to measure fGCM from 261 different individuals whose body conditions were scored (Supplementary Table 2).

### Extraction and analysis of hormonal metabolites

fGCMs were extracted from faecal samples and analysed according to the protocol given in Ganswindt *et al.* (2003, 2005a). The frozen stored samples were lyophilized, pulverized and sifted before extracting 0.05 g of each dried faecal powder using 3 ml of 80% ethanol. This solution was vortexed for 15 min followed by 15 min of centrifugation at 2500 *g* and 37°C. The supernatant was then decanted into microcentrifuge tubes and stored at −20°C until further analysis.

#### Enzyme immunoassay procedure and validation

The diluted extracts (1:20, 1:40 or 1:80 in aqueous assay buffer) were used for measuring immunoreactive fGCM (μg/g). We used the group-specific 11-oxoetiocholanolone enzyme immunoassay (EIA; lab-code: 72T with an antibody produced against 5β-androstane-3α-ol-11-one-17CMO:BSA and 5β-androstane-3α-ol-11,17-dione-17-CMO-biotinyl-3,6,9 trioxaundecanediamin as a label), which previously has been shown to provide reliable information on adrenocortical function in African elephants (Ganswindt *et al.*, 2003, 2005a, 2010a; Viljoen *et al.*, 2008) and has been validated in Asian elephants as well (Laws *et al.*, 2007; Ghosal *et al.*, 2013). Cross-reactivity of the used antibody is described in Möstl *et al.* (2002) and Huber *et al.* (2003). Concentration of fGCM was calculated from optical densities, obtained from a Tecan Infinite 200 PRO ELISA plate reader (with Magellan Software) at 450 nm, by using the sigmoidal dose response in GraphPad Prism 5. We analytically validated the EIA by measuring its sensitivity (0.9 pg/well at 90% binding) and by demonstrating parallelism between the serial dilution of pooled faecal extracts and the standards (Supplementary Figure 2). Intra- (*n* = 85) and inter-assay coefficients (*n* = 40) of variations of high- and low-value quality controls ranged between 3.9% and 9.4%.

### Statistical analyses

To represent the relationship between categorical factors such as season (dry and wet) and age classes with the BCS and also to visualize the annual changes in BCS, descriptive statistics (for instance, bar graphs) were used. Statistical difference between these factors was determined using Fisher's exact test in males (as some cells in the contingency table had sample sizes less than 5), and Monte Carlo simulations (based on 2000 replicates) in females (some of the cells had sample sizes less than 5 but the total count was too high for Fisher's exact test to be used). No non-independent observations (repetitive samples) were used for analysing the influence of seasonality on BCS. Repeat observations were randomly deleted, with a single sample retained, prior to Fisher's exact test and the significance tests were performed each time. The statistical results, however, remained unchanged in each set of data with randomly deleted repeat observations, thereby suggesting that the results are robust. To avoid the influence of sex on the analyses, female and male samples were analysed separately in all cases.

We used generalized linear models (GLMs) with gamma distributed errors (the errors were non-normally distributed and variance were non-constant) and the log link function to analyse the influence of BCS (predictor) on fGCM (continuous positive response variable). Besides BCS, we also used the interaction terms between BCS and season (dry and wet), sex (female and male) and age class (adult, sub-adult, juvenile and calf) to check their influence on hormone metabolite concentration. As the GLM results seem to be largely driven by the fGCM for BCS = 1, we also carried out post hoc Tukey's HSD (honest significant difference) test using the function ‘ghlt’ in the package ‘multcomp’ for the pair-wise comparisons between the mean fGCM levels for each BCS. In order to avoid anticonservative *P*-values and inflated goodness-of-fit, we report the full models without performing any stepwise model selection. As individuals with BCS 1 were infrequently sighted in the wild, there was unequal sample size in the BCS category leading to unbalanced design for understanding the influence of BCS on fGCM. However, simulations showed that the unbalanced design did not affect Type-I or Type-II error rates or the accuracy of coefficient estimates, though the precision of coefficient estimate is reduced, as expected, in the small sample case (Supplementary Figure 3). For the fGCM analysis, we had 223 non-repetitive faecal samples from 223 individuals and 84 repetitive faecal samples from 38 individuals. To check the influence of multiple samples of some individuals, we calculated the autocorrelation function of model residuals and found no evidence of statistically significant temporal autocorrelation (Supplementary Figure 4). We used R version 3.1.3 (R Core Team, 2015) for all analyses.

## Results

### Annual changes in BCS

**Figure 2:**
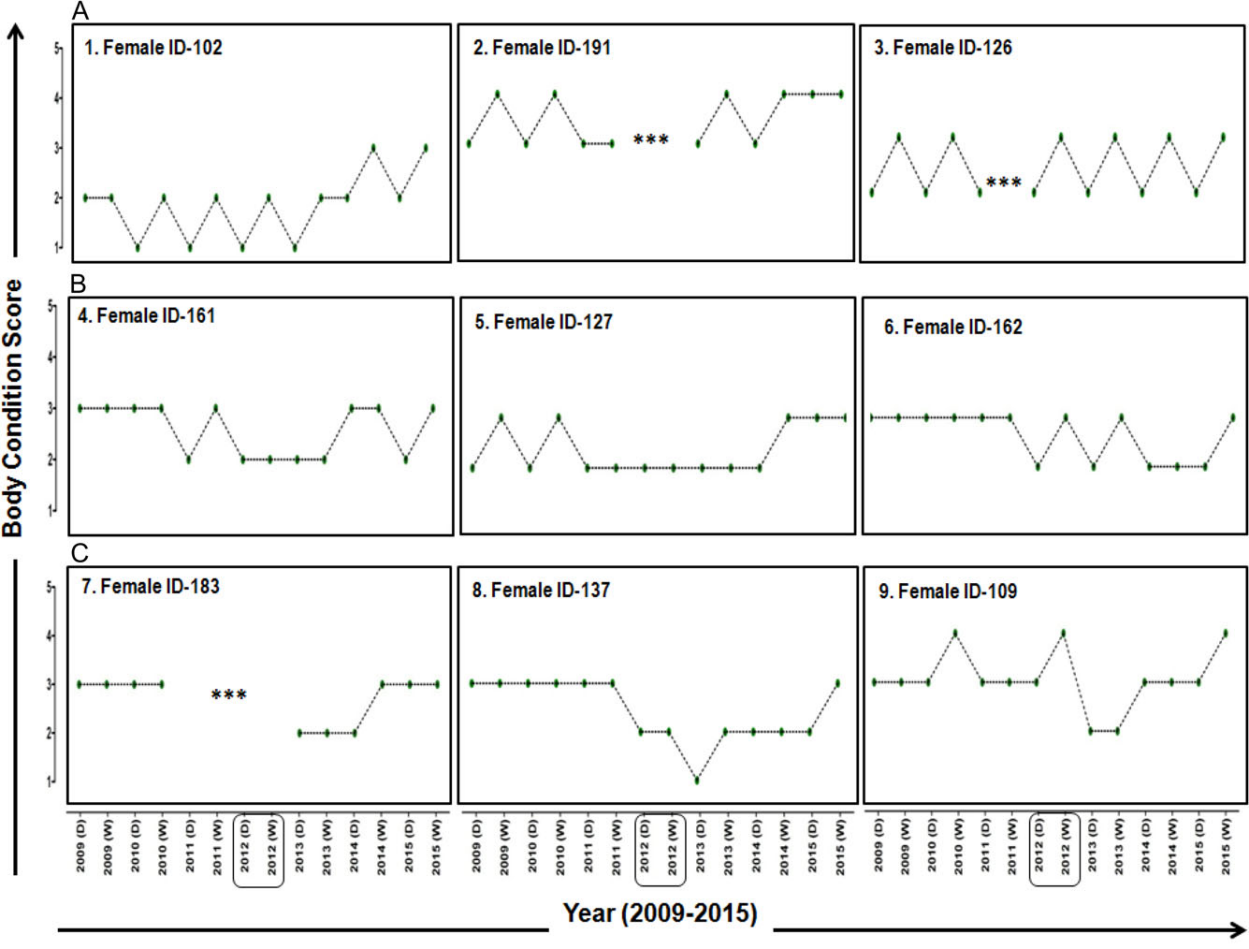
Changes in BCS of nine adult female elephants between dry and wet seasons from 2009 to 2015. The *X*-axis represents seasons (D = dry and W = wet) through the period 2009–2015, and the *Y*-axis represents BCS. Each plot represents changes in BCS for each adult female. Panels ‘A’, ‘B’ and ‘C’ represent individuals exhibiting similar profiles of change in BCS. Symbols represent: ***: not sighted; 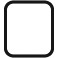: Drought year.

Changes in the BCS of nine adult female elephants in Mudumalai between dry and wet seasons during the seven year study period (2009–2015) are shown in Fig. 2. All (nine) animals showed low BCS during the dry season, while BCS were high during the wet season. Based on the similarity in profiles of change in BCS, animals were grouped into three different categories: individuals that showed constant pattern of seasonal fluctuations in their BCS between two seasons (Fig. 2, Panel A), individuals that showed a constant BCS for a certain period of time followed by seasonal fluctuations (Fig. 2, Panel B) and individuals that showed decline in their BCS (for instance, 8 and 9) followed by recovery (Fig. 2, Panel C).

### Season-dependent changes in BCS

**Figure 3:**
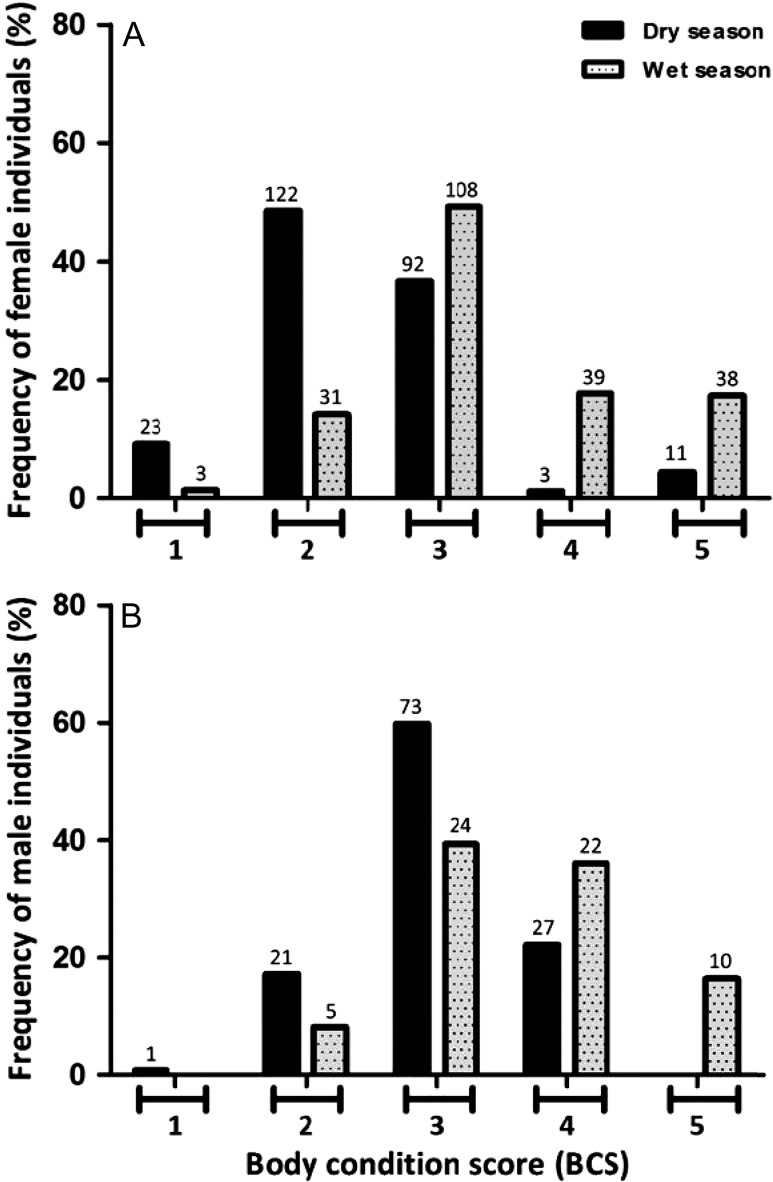
Proportion of elephants showing various BCS between dry and wet seasons. Bar plot representing: (**A**) frequency (%) of female individuals (*n* = 470) with different BCS in the two seasons; (**B**) frequency (%) of male individuals (*n* = 183) with different BCS in the two seasons. The number over each bar represents the sample size for that BCS (see text for statistical test results).

Changes in BCS between (a) dry and wet seasons and (b) early and late wet seasons were analysed. The influence of dry and wet seasons on the BCS of 653 unique elephants was evaluated. For both females (*n* = 470) and males (*n* = 183), the pattern was significantly skewed towards lower BCS during the dry season than in the wet season (for females: Monte Carlo simulations, *P* < 0.001; for males: Fisher's exact test, *P* < 0.001, Fig. 3). Similar profiles were observed for adult females (*n* = 353) and adult males (*n* = 131) between dry and wet seasons (Monte Carlo simulations and Fisher's exact test, *P* < 0.001, Supplementary Figure 5). To assess whether or not the differences in the intensity of rainfall influences the BCS of animals, we subdivided the wet season into early wet and late wet season. We observed significant difference in BCS values of female and male individuals between early and late wet season (Fisher's exact test, *P* < 0.001, Supplementary Figure 6).

### Association between BCS and fGCM

**Figure 4:**
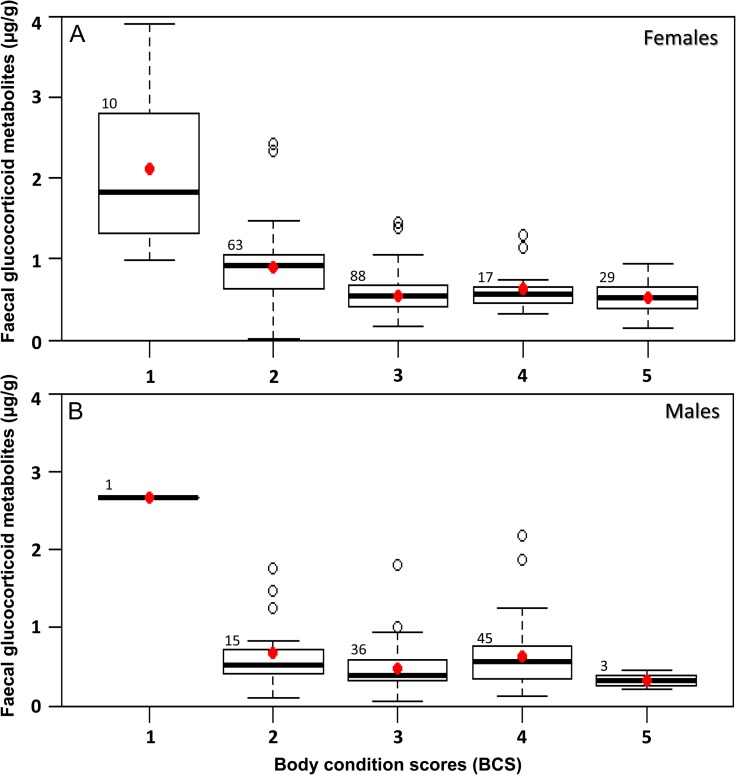
Association between BCS of elephants and their fGCMs levels. Box plots representing: (**A**) grouped concentrations of fGCM level (μg/g) of female individuals (*n* = 207) and (**B**) grouped concentrations of fGCM level (μg/g) of male individuals (*n* = 100) with BCS ranging from 1 to 5. The boxes show the median value and the upper and lower quartile values. The whiskers show the 10th and 90th percentiles of the values. Red dots represent the mean fGCM levels for the respective BCS. Numbers above the box represent sample sizes for each BCS. Statistically significant differences in fGCM concentrations of female individuals with different BCS, determined using the GLM and post hoc Tukey's HSD test, are explained in Supplementary Table 5.

There is a strong negative association between BCS and fGCM with a sharp decline in fGCM levels from BCS 1 to BCS 3 which remained almost similar between BCS 3, 4 and 5 in female individuals (GLM, *P* < 0.0001; Fig. 4A). From the GLM analysis for female individuals, the highest fGCM values (2.11 μg/g, SD = 1.03) were observed in individuals with BCS 1 while the lowest values of fGCM were observed in individuals with BCS 5 (0.53 μg/g, SD = 0.22) and the observed differences between fGCM content and BCS were found to be statistically significant (GLM, *P* < 0.0001; Table 1). When the mean fGCM levels of all female and adult female individuals were compared with different BCS, individuals with BCS 2 to BCS 5 had significantly lower fGCM when compared with individuals with BCS 1 (Tukey's HSD, *P* < 0.001); similarly, individuals with BCS 3 to BCS 5 had significantly lower fGCM than individuals with BCS 2 (Tukey's HSD, *P* < 0.05) but there were no significant differences when individuals with BCS 3, 4 and 5 were compared (Tukey's HSD, *P* > 0.05; Supplementary Table 5).

**Table 1:** Effect of BCSs, season, age class and their interaction with BCS on levels of fGCMs (*n* = 307) based on the GLM (gamma family, log link function)

Predictor variables	Level	Female^a^ (*n* = 207)				Male^b^ (*n* = 100)			
		Estimate	± SE	*t* value	Pr(>|*t*|)	Estimate	± SE	*t* value	Pr(>|*t*|)
	(Intercept)	**0.59**	**0.15**	**3.8**	**0.00016**	0.97	0.99	0.98	0.33
BCSs (1 to 5)									
	**BCS 2**	**−0.61**	**0.17**	**−3.57**	**0.00045**	−1.39	1.02	−1.37	0.17
	**BCS 3**	**−1.24**	**0.17**	**−7.21**	**1.30E-11**	−1.72	1	−1.72	0.09
	**BCS 4**	**−1.4**	**0.27**	**−5.16**	**6.15E-07**	−1.53	0.1	−1.53	0.13
	**BCS 5**	**−0.98**	**0.2**	**−4.84**	**2.73E-06**	−2.1	1.2	−1.75	0.08
Season (wet and dry)									
	Season (wet)	0.63	0.34	1.83	0.07	0.02	0.84	0.03	0.98
Age class (A, SA, J and C)									
	Age class (calf)	−0.05	0.44	−0.11	0.91	−0.82	0.81	−1.02	0.31
	Age class (juvenile)	−0.18	0.2	−0.86	0.39	−0.62	0.42	−1.47	0.14
	Age class (sub-adult)	−0.09	0.18	−0.52	0.6	0.13	0.71	0.02	0.98
BCS × season interaction									
	BCS 2 : Season (wet)	−0.68	0.36	−1.89	0.06	0.57	0.97	0.59	0.55
	BCS 3 : Season (wet)	−0.49	0.36	−1.38	0.17	0.26	0.88	0.3	0.77
	BCS 4 : Season (wet)	−0.4	0.42	−1.04	0.3	0.27	0.87	0.31	0.76
	**BCS 5 : Season (wet)**	**−1.06**	**0.38**	**−2.81**	**0.005**				
BCS × age class interaction									
	BCS 2 : Age class (calf)	−0.6	0.62	−0.97	0.33				
	BCS 2 : Age class (juvenile)	−0.11	0.33	−0.35	0.73	−0.07	0.91	−0.08	0.93
	**BCS 2 : Age class (sub-adult)**	**−1.04**	**0.3**	**−3.62**	**0.0004**	−0.58	0.84	−0.69	0.49
	BCS 3 : Age class (sub-adult)	0.02	0.21	0.08	0.93	−0.21	0.77	−0.27	0.79
	**BCS 4 : Age class (sub-adult)**	**1.02**	**0.35**	**2.96**	**0.0034**				

Statistically significant differences are in bold font.

^a^GLM (formula = Hormone ~ BCS + season + age class + BCS × season + BCS × age class, family = gamma(link = log), data = female).

^b^GLM (formula = Hormone ~ BCS + season + age class + BCS × season + BCS × age class, family = gamma(link = log), data = male).

There was no significant relationship between fGCM levels and BCS in male individuals, though there was a strong significant difference between fGCM levels of males with BCS 2, BCS 3, BCS 4 and BCS 5 when compared with BCS 1 (2.67 μg/g, GLM, *P* < 0.0001). As there was only one sub-adult male having BCS 1 this significance is not meaningful. Besides this, the pair-wise comparisons of mean fGCM levels in all males and adult males with different BCS using Tukey's HSD were not found to be significant.

When the interactions between BCS, season and age class were compared, the only significant differences in fGCM levels observed were the following: female individuals with BCS 5 (wet season) had lower levels than those with BCS 1 (dry season), sub-adult females with BCS 2 had lower levels than adult females with BCS 1, and sub-adult females with BCS 4 had higher levels than adult females with BCS 1 (Table 1).

To discount the influence of age class and sex on BCS and fGCM parameters, we performed separate GLMs including only adult females (*n* = 165, Supplementary Table 3) as well as adult males (*n* = 82, Supplementary Table 4) with interaction terms of BCS and season. For adult females, a similar pattern of significant decline in fGCM level with increasing scores from BCS 1 to BCS 2 and to BCS 3 was observed, with differences in fGCM being non-significant between BCS 3, BCS 4 and BCS 5 (Supplementary Figure 7A). Moreover, interaction between BCS and season showed significance only for BCS 2 and BCS 5 in the wet season in comparison to adult females having BCS 1 in the dry season (Supplementary Table 3). For adult males, no significant patterns were observed when BCSs were compared with fGCM, and no significant interaction between BCS and season was observed (Supplementary Figure 7B; Supplementary Table 4).

## Discussion

The body condition of free-ranging Asian elephants, inhabiting seasonally dry tropical forests, varies over season and years, especially in the case of female individuals. Similarly, both female and male individuals show a clear pattern of shift towards thinner body condition during the dry season as compared to the wet season, as could be expected and also suggested by several other studies on elephants (Foley *et al.*, 2001; Ganswindt *et al.*, 2010b; Ramesh *et al.*, 2011; Mumby *et al.*, 2015). Observations from annual changes in visual BCS showed that free-ranging Asian female elephants go through varied trends of fluctuation over the years; no studies have been conducted in the wild to evaluate the yearly changes in BCS so far. As could be expected, the occurrence of drought (2012 was declared as a drought year by the India Meteorological Department and its environmental impact carried over into the dry season of the following year when we sampled the population) negatively influences BCS. A study of the world's largest semi-captive population of Asian elephants employed in the timber industry in Myanmar showed that body condition (weight) is positively correlated to rainfall and inversely correlated to the population mortality rates one month later (Mumby *et al.*, 2015).

A seasonal shift in the diet of elephants from high-quality grasses (higher crude protein) in the wet season to poorer quality grass, and sparsely available green leaves and twigs during the dry season (Western and Lindsay, 1984; Sukumar, 1989; Campos-Arceiz *et al.*, 2008) could explain the decline in BCS during the dry period. Additionally, a study on faecal nitrogen as an indicator of nutritional status found that the percentage of faecal nitrogen increased from dry to wet season in African elephants implying seasonal changes in nutritional quality (Codron *et al.*, 2006). In our study, we too observed a seasonal pattern of change in BCS which may be explained by changing energy requirements and resource availability (access to quality food and water; Turner *et al.*, 2012).

Apart from seasonal fluctuations in quality and quantity of resources, other factors such as gastrointestinal parasites and nematodes in elephants are responsible for several illnesses, poor body condition and even mortality (Vidya and Sukumar, 2002; Kinsella *et al.*, 2004; Irvine, 2006; Obanda *et al.*, 2011; Thurber *et al.*, 2011; Baines *et al.*, 2015). In this study, we did not assess the parasite loads (although some of the adult males and females had visible parasites, mostly nematodes, in their dung) that may increase during the dry season and influence the BCS (Vidya and Sukumar, 2002). However, Obanda *et al.* (2011) and Turner *et al.* (2012) suggested a synergistic consequence of parasitism and severe climatic conditions such as drought, resulting in host starvation and dehydration, may cause intestinal tissue abrasion, malnutrition, emaciation and ultimately, death. Additionally, environmental variability has also been recognized as an important factor affecting host stress and immunocompetence (Altizer *et al.*, 2006). Hence, one of the major factors that might be defining seasonal change in BCS is the quality of food resources (Van Soest, 1994; Rode *et al.*, 2006; Turner *et al.*, 2012). Prolonged exposure to stressful conditions such as resource limitation, parasite loads and nutrient-deficient diet during the dry season may elevate glucocorticoid levels which could lead to excessive protein catabolism and muscle deterioration, ultimately resulting in poorer body condition (Harvey *et al.*, 1984). A seasonal shift in BCS from lower to higher condition between dry to wet seasons could also suggest the redeployment of fat reserves between seasons.

Our study also showed a strong negative association between BCS and fGCM. We found that the hormone metabolite levels declined with an increase in the BCS from 1 to 3. Although cause and effect remain unclear, several other studies also report a negative relationship of fGCM with body condition in birds, amphibians and reptiles, thereby emphasizing the role of glucocorticoid hormones in energy mobilization (Moore and Jessop, 2003; Husak and Moore, 2008; Gladbach *et al.*, 2011). Cortisol or glucocorticoid is a gluconeogenic hormone which produces glucose by promoting the breakdown of muscle, bone, and connective tissues; thus, elevated levels of cortisol during stress will break down these structural tissues resulting in inhibition of protein synthesis, elevated energy mobilization, and reduction in body mass (Kitaysky *et al.*, 1999; Landys *et al.*, 2006; Cabezas *et al.*, 2007; Schakman *et al.*, 2013). Ganswindt *et al.* (2010b) also found that, with an elevated level of fGCM, the BCS declined in two physically injured African elephants.

There was a general lack of interaction between BCS, season and age class in relation to fGCM; however, the higher fGCM levels in female individuals with BCS 1 (dry season) than those with BCS 5 (wet season) are in tune with the expectations from lower resource availability during the dry months. Similarly, sub-adult females with BCS 2 and BCS 4 showing significant differences in fGCM levels than adult females; this could be because of sexual maturation or physiological challenges such as pregnancy and lactation experienced by the latter.

When we segregated the data to discount the influence of age class and sex and compared only adult females and adult males separately, we found that adult females exhibited a clear decline in fGCM with increase in body condition, from score-1 to score-3, unlike adult males where no such trend was observed. One of the possible reasons for a clear and significant pattern of elevated level of fGCM in females with declining BCS may be their reproductive status (for example, lactating females with calves might be more susceptible to physiological or nutritional stress) or their social responsibilities in the herd (Moss and Poole, 1983). The lack of clear patterns in mean fGCM levels among male elephants with different BCS may be due to several reasons. In polygynous mammals such as elephants, adult males are generally solitary and attain larger body-size than females (Laws, 1966; Sukumar *et al.*, 1988; Poole, 1989; Chiyo *et al.*, 2011). Males show prolonged growth in stature and body mass throughout their life (Laws, 1966; Sukumar *et al.*, 1988). On the other hand, they undergo frequent physiological (musth) and behavioural changes due to intense reproductive competition for very scarce and highly mobile reproductive females (Roth, 1984; Hollister-Smith *et al.*, 2007; Chelliah and Sukumar, 2013). Consequently, BCS among male elephants are also subject to these varying energetic demands. Male elephants are known to meet their increased energy demands through spatial and sexual segregation by ranging in exclusive ‘bull areas’ (Poole, 1987; Shannon *et al.*, 2006) and in agricultural fields (Sukumar and Gadgil, 1988; Chiyo *et al.*, 2011). This may be one of the reasons why we found very few male individuals with the lowest BCS. The lower average hormone metabolite levels in males (0.60 μg/g ± 0.06) than in females (0.74 μg/g ± 0.03) could also be because of a delayed suppressive effect of musth on adrenocortical endocrine function, in some of the adult males we sampled, as suggested by Ganswindt *et al.* (2003, 2005a, 2010a).

The present study did not show any significant relationship between fGCM and seasons, unlike earlier studies which demonstrated elevated levels of fGCM during the dry season in African elephants (Foley *et al.*, 2001; Rasmussen *et al.*, 2008; Viljoen *et al.*, 2008; Ganswindt *et al.*, 2010b) and seasonal effects on reproductive and stress hormones (salivary cortisols) of semi-captive and captive Asian elephants (Menargues *et al.*, 2008; Thitaram *et al.*, 2008). Although there was a declining trend in fGCM with increasing BCS between dry and wet seasons, the difference between seasons was not significant. We also did not find any significant difference with age class when observed either combined with BCS or independently. A similar relation was also observed by Viljoen *et al.* (2008) and Ganswindt *et al.* (2005b) who too reported no significant influence of age class on the fGCM level.

Although a short-term stress response may be beneficial to an organism, long-term exposure to stress may adversely impact its health, reproduction and longevity (von Holst, 1998; Moberg, 2000). Multiple physiological, social, behavioural and ecological factors including metabolic rate, social structure, dominance rank, reproductive status, diet type, gut microbes, parasite loads and diseases synergistically influence the stress status of free-ranging animals (Wingfield *et al.*, 1998; Koolhaas *et al.*, 1999; Cockrem, 2013; Dantzer *et al.*, 2014; Haase *et al.*, 2016). While an animal in extremely ‘emaciated’ body condition due to starvation or disease is obviously at high risk of mortality and, at the other end of the body condition scale, an ‘obese’ individual likewise would be considered unhealthy and prone to higher risk of death (in humans and many other mammal species), the relationship between intermediate body types and fitness of an individual is still an open question. Calorie restriction (and possibly a body condition tending towards ‘lean’) is recognized as a strong correlate of longevity in humans, nonhuman primates and rodents (Merry, 2002; Bordone and Guarente, 2005). In tune with seasonal cycles, wild animals go through periods of deprivation and plenty. However, without an understanding of the relationship between body condition and measures of fitness, the use of terms such as ‘normal’, ‘optimum’ or ‘ideal’ to describe elephant body types could perhaps be best avoided in a biological context.

In conclusion, we have demonstrated that physical body condition of elephants is season-dependent and this change is reflected in fGCM levels as well. Our study is among the first to explore the influence of seasonality on BCS and its relationship to stress status (as determined by measuring cortisol levels) in a free-ranging elephant population. Yet, we still lack an understanding of the influence of stress levels (or more specifically cortisol levels) on the fitness of elephants. Such an understanding would require longitudinal studies on elephant populations in different situations of habitat type, seasonality and stress levels, by comparing across gradients of fragmentation and anthropogenic disturbances. This could contribute to planning for the welfare, conservation and management of free-ranging elephant populations.

## Supplementary Material

Supplementary Data

Supplementary Data
