# Supplementary material for: Assessment of season-dependent body condition scores in relation to faecal glucocorticoid metabolites in free-ranging Asian elephants
Source: Conserv Physiol. 2017 Jun 27;5(1):cox039. doi: 10.1093/conphys/cox039 (PMC5508666; doi:10.1093/conphys/cox039)
Supplement: Supplementary Data [file SupplementaryFigures.docx]

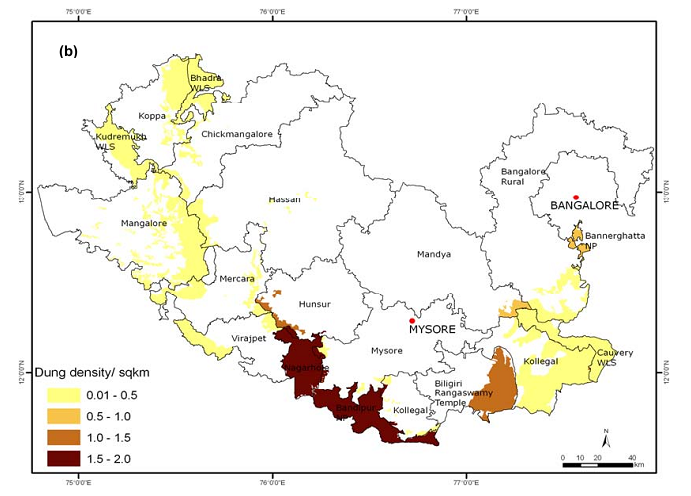

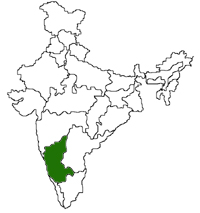

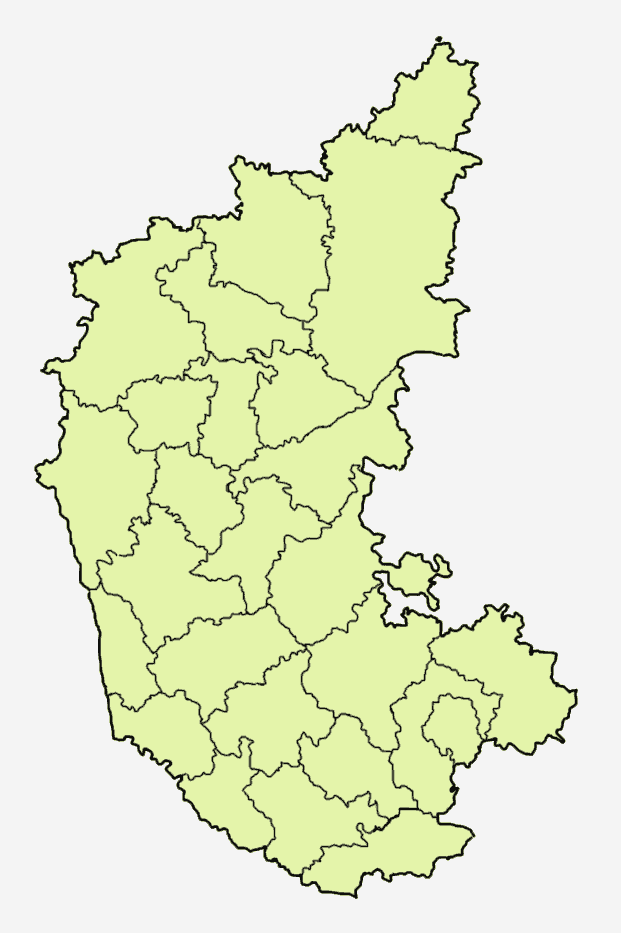


**B**=Bandipur National Park

**N**=Nagarhole National Park

**H**=Hassan

**B**

**N**

**H**

**Supplementary Figure 1:** Distribution map of elephants showing the main study sites in the state of Karnataka, India. Relative elephant densities are also shown based on the Karnataka Elephant Census – 2010.


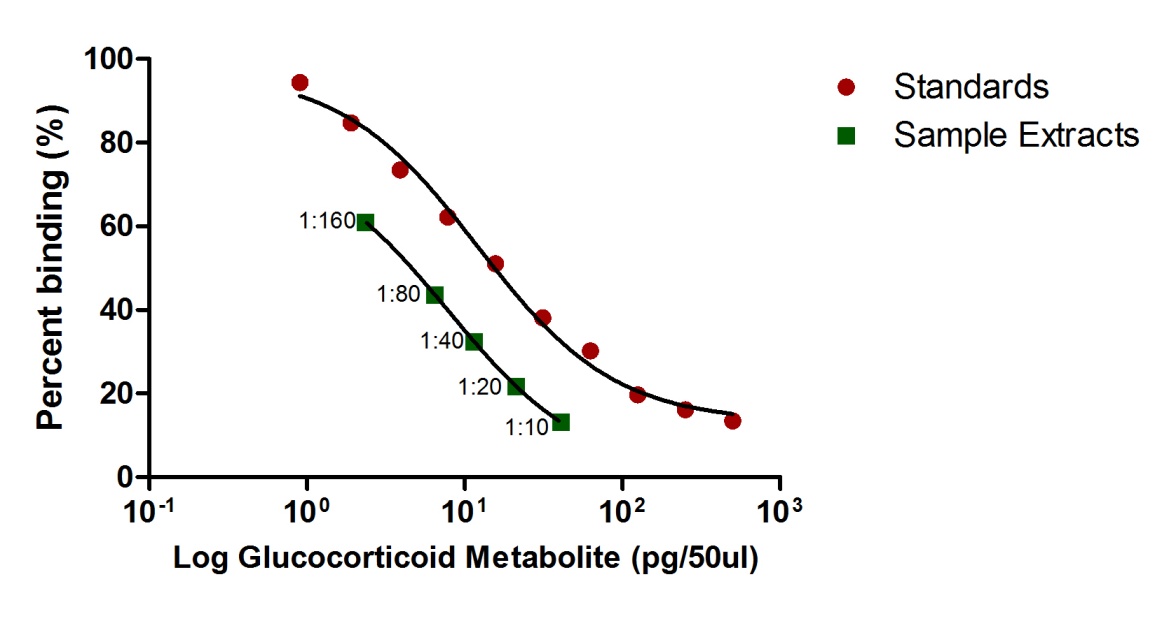


**Supplementary Figure 2:** Graph showing parallelism between serial dilutions (ratios starting from 1:10 to 1:160) of a faecal extract from an elephant (green square) and standards (red circle) of the 11-oxoetiocholanolone EIA.


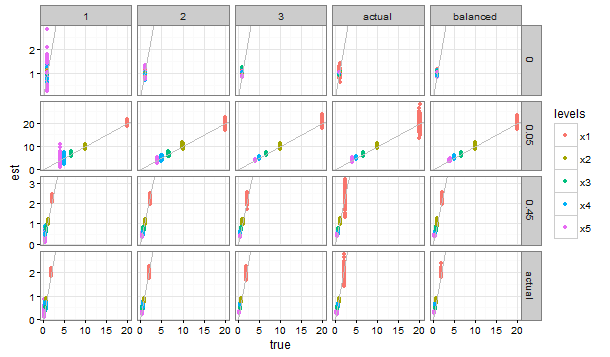


**A**


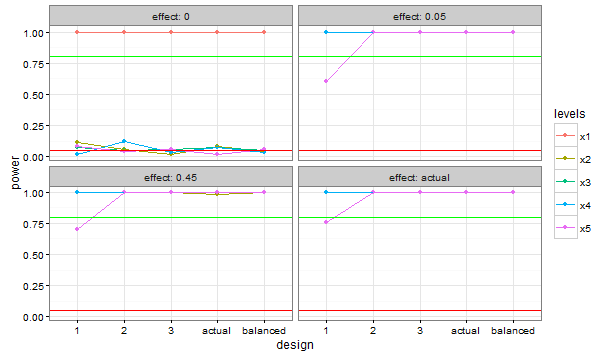


**B**

**Supplementary Figure 3:** The influence of unbalanced designs on the Type I error rate, power, accuracy and precision (the simulation study (number of simulations=100) with a total of 300 observations and five factor levels of a predictor variable (x1 to x5) was performed). The simulation included five different designs; a balanced design, actual data set and three unbalanced designs with the value indicating the degree of unbalancing (3 minimum, 1 maximum). Similarly, different effect sizes were chosen where effect size 0 reflected the null hypothesis and the final effect size was based on the actual data set). (A) Plots of accuracy (true vs. estimated regression coefficients) where each box in the row represents different designs with same effect size. (B) Influence of unbalanced designs on power with different effect sizes.


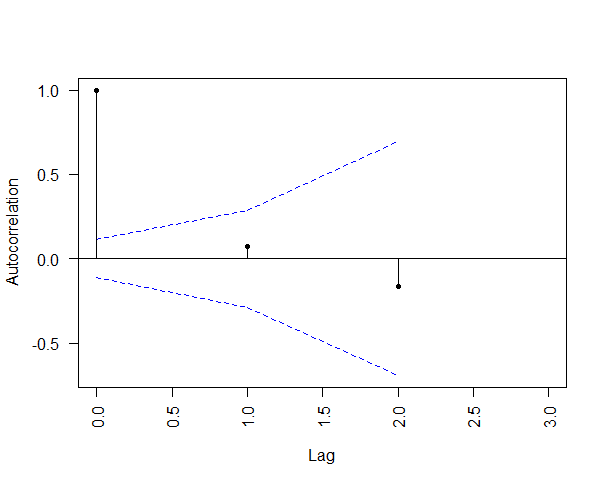


**Supplementary Figure 4:** Autocorrelation function (ACF) of model residuals to check the influence of the repetitive samples. No evidence of statistically significant temporal autocorrelation was observed.


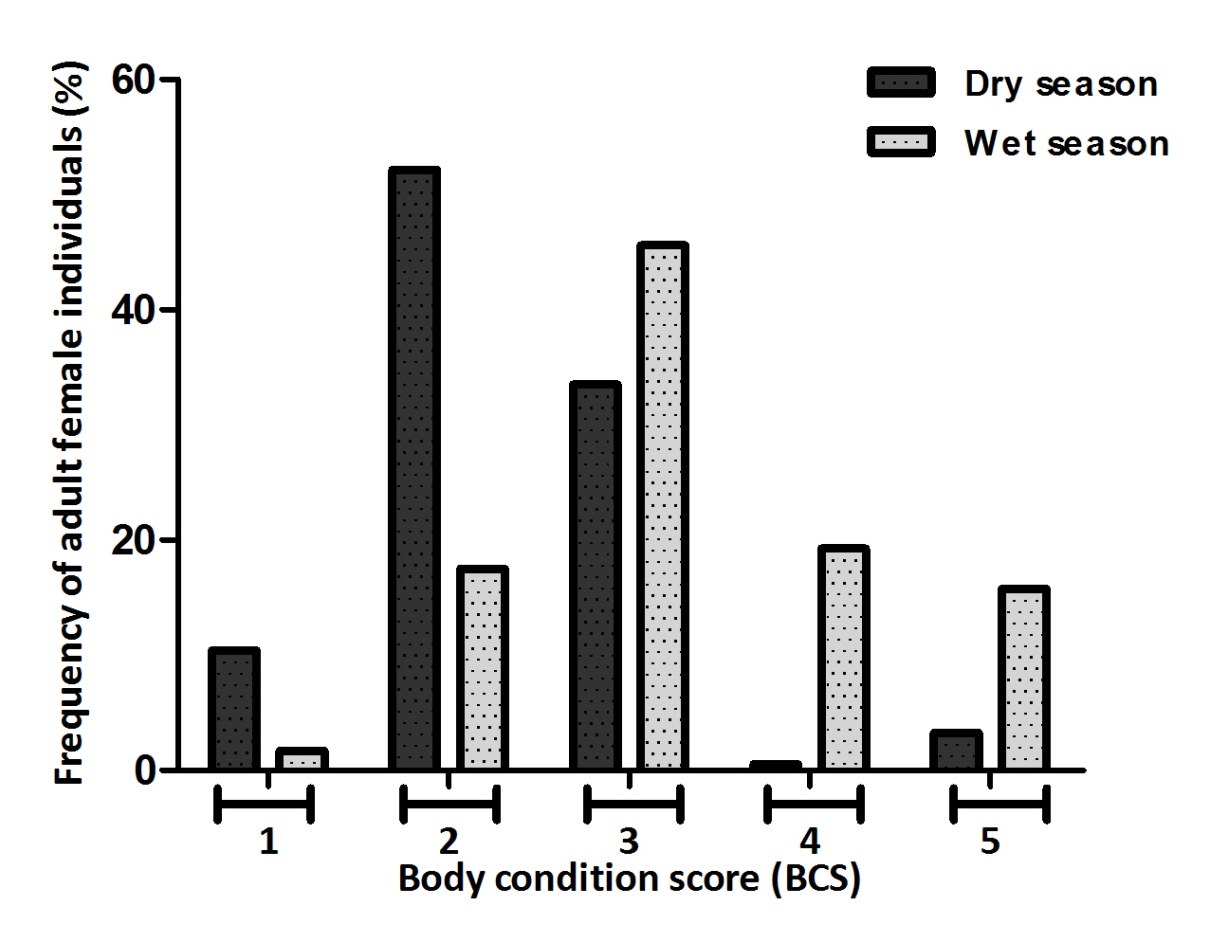

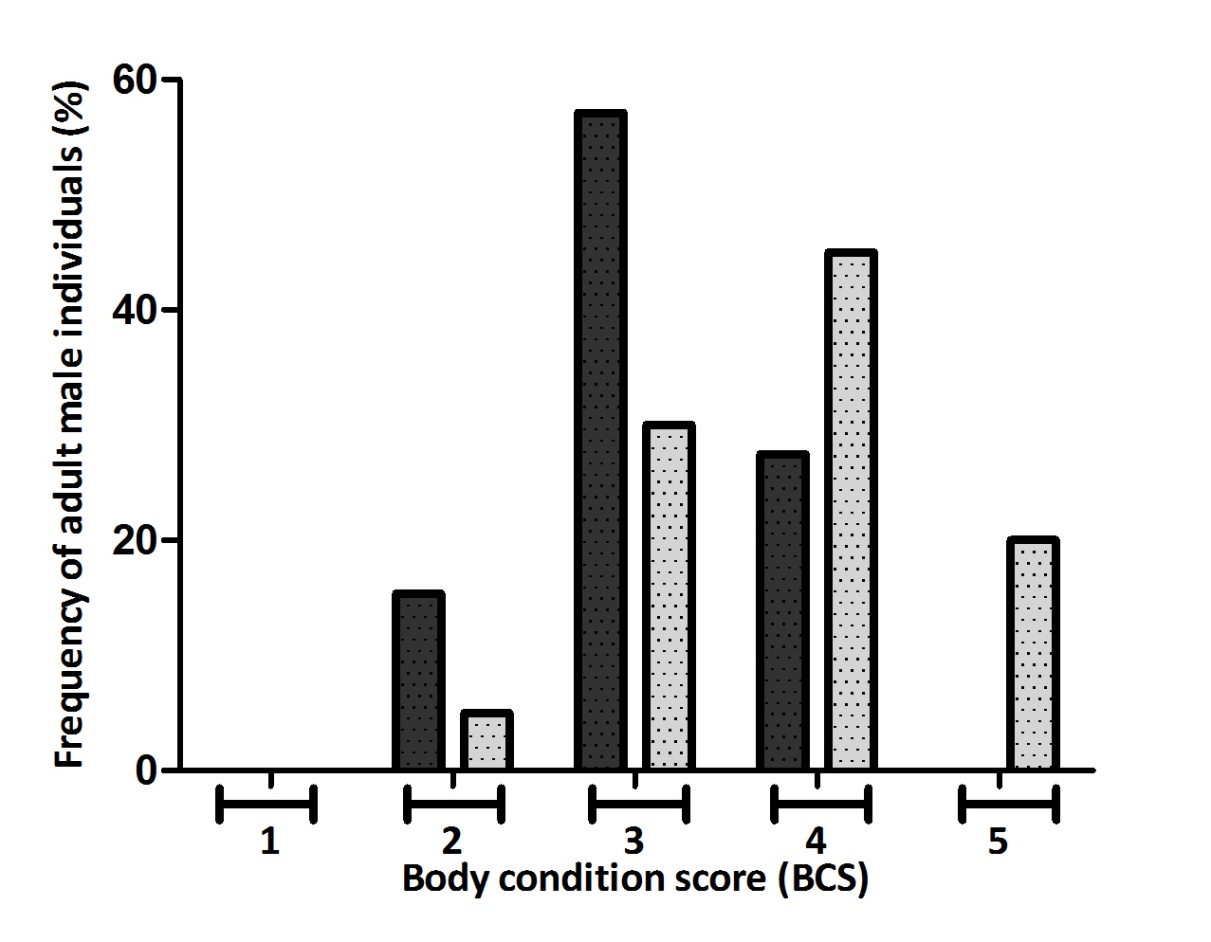


**A**

**B**

14

25

2

18

8

19

3

95

78

61

1

6

30

33

27

52

12

**Supplementary Figure 5:** Proportion of elephants showing various BCS between dry and wet seasons. Bar plot representing: A) Frequency (%) of adult female individuals (n = 353) with different BCS in the two seasons and B) Frequency (%) of adult male individuals (n=131) with different BCS in the two seasons. The number over each bar represents the sample size for that BCS (see text for statistical test results).


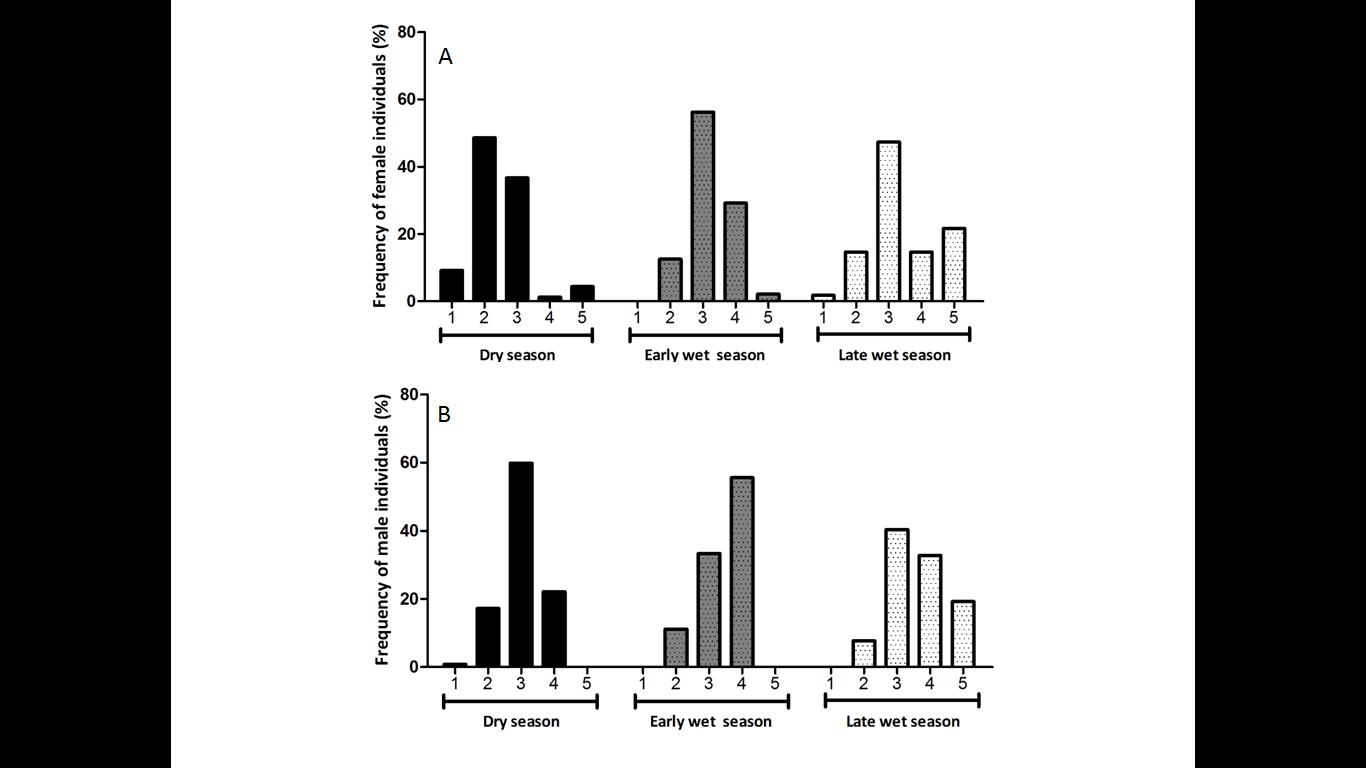


**Supplementary Figure 6:** Change in body condition scores (BCSs) between dry, early wet and late wet season. Bar graphs representing: A) The number of female individuals showing a seasonal shift in BCS between dry (n=251), early wet (n=48) and late wet (n=171) and B) The number of male individuals showing a seasonal shift in BCS between dry (n=122), early wet (n=9) and late wet (n=52).


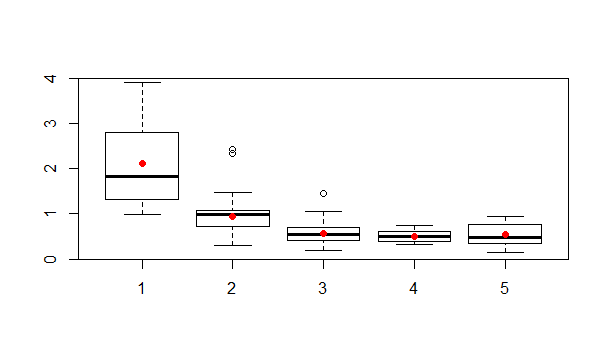


10

55

65

14

21

**1**

**2**

**3**

**4**

**0**

**Faecal glucocorticoid metabolites (µg/g)**

**1**

**2**

**3**

**4**

**5**

**Body condition scores (BCS)**

Adult females


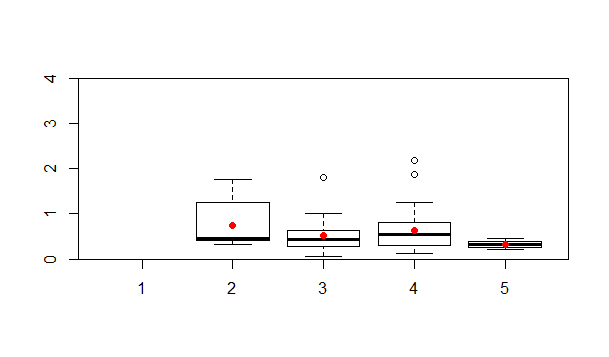


**1**

**2**

**3**

**4**

**0**

**Faecal glucocorticoid metabolites (µg/g)**

**1**

**2**

**3**

**4**

**5**

**Body condition scores (BCS)**

10

25

3

44

Adult males

**A**

**B**

**Supplementary Figure 7:** Association between BCS of elephants and their faecal glucocorticoid metabolites (fGCM) levels. Box plots representing: A) Grouped concentrations of fGCM level (µg/g) of adult female individuals (n = 165) and B) Grouped concentrations of fGCM level (µg/g) of male individuals (n = 82) with BCS ranging from 1 to 5. The boxes show the median value and the upper and lower quartile values. The whiskers show the 10^th^ and 90^th^ percentiles of the values. Red dots represent the mean fGCM levels for the respective BCS. Numbers above the box represent sample sizes for each BCS. Statistically significant differences in fGCM concentrations of adult female individuals with different BCS, determined using the Generalized Linear Model and post-hoc Tukey’s HSD test, are explained in Supplementary Table 5.
