# Supplementary material for: Assessment of season-dependent body condition scores in relation to faecal glucocorticoid metabolites in free-ranging Asian elephants
Source: Conserv Physiol. 2017 Jun 27;5(1):cox039. doi: 10.1093/conphys/cox039 (PMC5508666; doi:10.1093/conphys/cox039)
Supplement: Supplementary Data [file SupplementaryTables.docx]

**Supplementary Table 1.** Body condition scoring index for the Asian elephants (modified/ adapted from Morfeld et. al., 2016).

| **BCS** | **Descriptive term** | **Key body areas** | **Descriptive parameters** |
| --- | --- | --- | --- |
| **1** | **Very thin** | Ribs | Individual ribs clearly visible |
|  |  | Pelvic bone | Protrudes, deep depression in front and behind pelvic bone |
|  |  | Backbone | Protrudes from tail head to shoulders, deep depression alongside entire backbone |
|  |  |  |  |
| **2** | **Thin** | Ribs | Visible, covered by a very thin layer of fat |
|  |  | Pelvic bone | Clearly visible, obvious depression in front and/or behind pelvic bone |
|  |  | Backbone | Prominent from tail head to shoulders, obvious depression alongside entire backbone |
|  |  |  |  |
| **3** | **Medium** | Ribs | Slightly visible, covered by a layer of fat |
|  |  | Pelvic bone | Visible as a ridge, slight depression in front pelvic bone |
|  |  | Backbone | Clearly visible from tail head to mid-back, fat beginning to accumulate alongside backbone |
|  |  |  |  |
| **4** | **Fat** | Ribs | Not visible |
|  |  | Pelvic bone | Not entirely visible, slight sunken or flattened area in front of pelvic bone |
|  |  | Backbone | Visible as a ridge, visible than others due to fat accumulation |
|  |  |  |  |
| **5** | **Very fat** | Ribs | Not visible |
|  |  | Pelvic bone | Not visible |
|  |  | Backbone | Not visible; clear signs of fat deposition near neck and belly region |

**Supplementary Table 2.** Total number of elephants and dung samples collected for each analysis. A) 307 faecal samples were collected from 261 individuals (scored for body condition) for steroid analysis; B) 466 BCS samples (no faecal samples; only scored for body condition) were collected from 392 individuals and C) 773 BCS samples (samples from both A and B) were collected from 653 individuals for seasonal variation in BCS in two sampling years from Bandipur, Nagarahole and Hassan.

| **Sample size for each analysis** | **Total individuals (samples)** | **Unique individuals & samples** | **Repetitive individuals** |
| --- | --- | --- | --- |
| A. Relationship between fGCM and BCS analysis | 261 (307) | 223 | 38 |
| B. BCS assessed (no faecal sample collected) | 392 (466) | 329 | 63 |
| C. Total available samples (A+B) | 653 (773) | 552 | 101 |
| D. Relationship between BCS and season analysis | 653 (653) | 552 | 101 |

**Supplementary Table 3.** Effect of body condition scores (BCS), season and their interaction on levels of faecal glucocorticoid metabolites level (n=165) of adult females based on the Generalized Linear Model* (Gamma family, log link function). Statistically significant differences are in bold font.

| **Predictor variables** | **Level** | **Estimate** | **± SE** | **t value** | **Pr(>\|t\|)** |
| --- | --- | --- | --- | --- | --- |
| **Intercept** |  | **0.59** | **0.14** | **4.08** | **7.2E-05** |
| **Body condition score (BCS)** |  |  |  |  |  |
|  | **BCS2** | **-0.62** | **0.16** | **-3.79** | **0.00021** |
|  | **BCS3** | **-1.24** | **0.16** | **-7.52** | **4.1E-12** |
|  | **BCS4** | **-1.43** | **0.28** | **-5.20** | **7.4E-07** |
|  | **BCS5** | **-0.92** | **0.20** | **-4.64** | **7.5E-06** |
| **Season** |  |  |  |  |  |
|  | Season (Wet) | 0.63 | 0.32 | 1.94 | 0.06 |
| **BCS*Season interaction** |  |  |  |  |  |
|  | **BCS(2): Season (Wet)** | **-0.67** | **0.34** | **-1.97** | **0.05** |
|  | BCS(3): Season (Wet) | -0.49 | 0.34 | -1.45 | 0.15 |
|  | BCS(4): Season (Wet) | -0.42 | 0.42 | -1.01 | 0.31 |
|  | **BCS(5): Season (Wet)** | **-1.16** | **0.37** | **-3.15** | **0.002** |

* glm(formula = Hormone ~ BCS + Season + BCS * Season, family = Gamma(link = log), data = Adult females)

**Supplementary Table 4.** Effect of body condition scores (BCS), season and their interaction on levels of faecal glucocorticoid metabolites level (n=82) of adult males based on the Generalized Linear Model (Gamma family, log link function). No significant variables and interaction was observed.

| **Predictor variables** | **Level** | **Estimate** | **± SE** | **t value** | **Pr(>\|t\|)** |
| --- | --- | --- | --- | --- | --- |
| **Intercept** |  | -0.39 | 0.25 | -1.56 | 0.12 |
| **Body condition score (BCS)** |  |  |  |  |  |
|  | BCS3 | -0.35 | 0.30 | -1.17 | 0.25 |
|  | BCS4 | -0.16 | 0.28 | -0.58 | 0.56 |
|  | BCS5 | -0.74 | 0.75 | -0.98 | 0.33 |
| **Season** |  |  |  |  |  |
|  | Season (Wet- Dry) | 0.44 | 0.56 | 0.77 | 0.44 |
| **BCS*Season interaction** |  |  |  |  |  |
|  | BCS(3): Season (Wet) | -0.16 | 0.65 | -0.25 | 0.81 |
|  | BCS(4): Season (Wet) | -0.15 | 0.61 | -0.24 | 0.81 |
|  | BCS(5): Season (Wet) | -0.42 | 1.04 | -0.40 | 0.69 |

* glm(formula = Hormone ~ BCS + Season + BCS * Season, family = Gamma(link = log), data = Adult males)

**Supplementary Table 5.** Pairwise comparisons of mean fGCM levels between five factor levels of BCS of all female and adult female individuals analyzed using Tukey’s HSD (function **ghlt** in the package **multcomp** in R-software). Statistically significant differences are in bold font.

| **Predictor variables** | **Levels** | **Females** | | | | **Adult females** | | | |
| --- | --- | --- | --- | --- | --- | --- | --- | --- | --- |
|  | **Comparisons of Means** | **Estimate** | **± SE** | **z value** | **Pr(>\|z\|)** | **Estimate** | **± SE** | **z value** | **Pr(>\|z\|)** |
| **BCS (1 to 5)** |  |  |  |  |  |  |  |  |  |
|  | **BCS (2-1)** | **-0.86** | **0.15** | **-5.66** | **<0.001** | **-0.8** | **0.14** | **-5.62** | **<0.001** |
|  | **BCS (3-1)** | **-1.34** | **0.15** | **-8.97** | **<0.001** | **-1.32** | **0.14** | **-9.37** | **<0.001** |
|  | **BCS (4-1)** | **-1.21** | **0.18** | **-6.8** | **<0.001** | **-1.42** | **0.17** | **-8.31** | **<0.001** |
|  | **BCS (5-1)** | **-1.38** | **0.16** | **-8.42** | **<0.001** | **-1.35** | **0.16** | **-8.51** | **<0.001** |
|  | **BCS (3-2)** | **-0.47** | **0.07** | **-6.44** | **<0.001** | **-0.52** | **0.07** | **-6.84** | **<0.001** |
|  | **BCS (4-2)** | **-0.35** | **0.12** | **-2.87** | **<0.05** | **-0.62** | **0.12** | **-5.05** | **<0.001** |
|  | **BCS (5-2)** | **-0.52** | **0.1** | **-5.16** | **<0.001** | **-0.55** | **0.11** | **-5.22** | **<0.001** |
|  | BCS (4-3) | 0.12 | 0.12 | 1.06 | 0.82 | -0.11 | 0.12 | -0.88 | 0.90 |
|  | BCS (5-3) | -0.04 | 0.09 | -0.44 | 0.99 | -0.03 | 0.1 | -0.34 | 0.10 |
|  | BCS (5-4) | -0.17 | 0.14 | -1.23 | 0.72 | 0.07 | 0.14 | 0.5 | 0.97 |
